# Use of TLC-Densitometric Method for Determination of Valproic Acid in Capsules

**DOI:** 10.3390/molecules27030752

**Published:** 2022-01-24

**Authors:** Wioletta Parys, Alina Pyka-Pająk

**Affiliations:** Department of Analytical Chemistry, Faculty of Pharmaceutical Sciences in Sosnowiec, Medical University of Silesia in Katowice, Jagiellońska 4, 41-200 Sosnowiec, Poland

**Keywords:** valproic acid, TLC, densitometry, copper sulfate, 2′,7′-dichlorofluorescein-aluminum chloride-iron (III) chloride, validation

## Abstract

Determination of valproic acid in the drug was carried out on the aluminum silica gel 60F_254_ plates and using acetone–water–chloroform–ethanol–ammonia at a volume ratio of 30:1:8:5:11 as the mobile phase, respectively. Two methods of detection of valproic acid were used. The first was a 2% aqueous CuSO_4_×5H_2_O solution, and the second was a 2′,7′-dichlorofluorescein-aluminum chloride-iron (III) chloride system. The applied TLC-densitometric method is selective, linear, accurate, precise, and robust, regardless of the visualizing reagent used for the determination of valproic acid in Convulex capsules. It has low limits of detection (LOD) and limits of quantification (LOQ), which are equal to 5.8 μg/spot and 17.4 μg/spot using a 2% aqueous CuSO_4_×5H_2_O solution as visualizing agent and also 0.32 μg/spot and 0.97 μg/spot using a 2′,7′-dichlorofluorescein-aluminum chloride-iron (III) chloride system as visualizing reagent, respectively. The described analytical method can additionally be used to study the identity of valproic acid in a pharmaceutical preparation. The linearity range was found to be 20.00–80.00 μg/spot and 1.00–2.00 μg/spot for valproic acid detected on chromatographic plates using a 2% aqueous CuSO_4_×5H_2_O solution and the 2′,7′-dichlorofluorescein-aluminum chloride-iron (III) chloride system, respectively. A coefficient of variation that was less than 3% confirms the satisfactory accuracy and precision of the proposed method. The results of the assay of valproic acid equal 96.2% and 97.0% in relation to the label claim that valproic acid fulfill pharmacopoeial requirements. The developed TLC-densitometric method can be suitable for the routine analysis of valproic acid in pharmaceutical formulations. The proposed TLC-densitometry may be an alternative method to the modern high-performance liquid chromatography and square wave voltammetry in the control of above-mentioned substances, and it can be applied when other analytical techniques is not affordable in the laboratory.

## 1. Introduction

**Figure 1:**
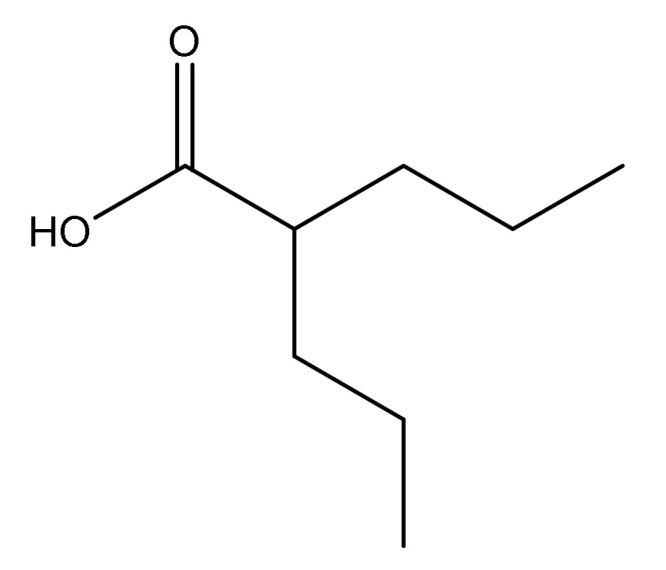
Chemical structure of valproic acid.

Valproic acid (Figure 1) and its salts are drugs with anticonvulsant activity used in the treatment of severe neurological diseases. They have also found application in the treatment of mania in bipolar disorder [1]. Valproic acid alleviates central nervous system (CNS) injuries and improves functional results after acute CNS damage by multiple pathways, including anti-inflammatory, anti-apoptotic, and neurotrophic effects [2]. Recent reports indicate that valproic acid can be used in the treatment of breast cancer [3] and in chronic lymphocytic leukemia [4].

Analytical methods are applied to determine the drug in human body fluids and in pharmaceutical preparations. They are useful in monitoring the therapeutic concentration of a substance, treatment progress, and side effects. Several methods for determination of valproic acid are known, namely: thin layer chromatography [5], liquid chromatography [6,7,8,9], high performance liquid chromatography [10,11,12,13,14,15,16,17,18,19,20,21,22,23,24], ultra high performance liquid chromatography [4,25,26], ultra performance liquid chromatography [27,28,29,30,31], gas chromatography [4,18,32,33,34,35,36,37,38], capillary electrophoresis [39,40], voltamperometry [41], valproate-selective electrode [42], and colorimetry [43].

The vast majority of valproic acid was determined in biological samples [4,5,6,7,8,9,10,11,12,13,16,19,20,21,22,24,25,26,27,28,29,30,31,32,33,34,35,36,38,39,40]. Only a few studies report the determination of valproic acid in pharmaceutical preparations [14,17,20,23,35,37,41,42]. From the analytical point of view, the methods that do not require derivatization of valproic acid [6,7,8,9,10,11,16,17,18,19,20,22,25,26,27,28,29,33,34,35,36,37,38,39,40,41,42] seem to be important because it shortens the time of the analysis. However, very often, pre-chromatographic derivatization of valproic acid allows the obtainment of lower values of its limit of detection. Abualhasan et al. [15] obtained the lowest value of the limit of detection among all publications discussed in this work. These studies were not concerned with the determination of sodium valproate in the matrix, but were model studies. Sodium valproate was pre-chromatographically derivatized. In the proposed method, the limit of detection for sodium valproate was 4.48 × 10^−9^ mol/dm^3^, whereas among the determination of valproic acid in biological matrices, Kamalinia et al. [24] obtained the lowest value of the limit of detection after valproic acid derivatization, which was 10 ng/mL (7 × 10^−8^ mol/dm^3^) during its determination in human plasma using the HPLC technique. In turn, in pharmaceutical matrices, during the determination using the HPLC technique with coulometric electrochemical detection of derivatized valproic acid in pharmaceutical preparations, its LOD was 0.75 pmol/mL (0.75 nmol/dm^3^) [14]. In the other methods cited above, the LOD of valproic acid ranged from 0.026 µg/mL to 85 µg/mL (from 1.8 × 10^−7^ mol/dm^3^ to 5.89 × 10^−4^ mol/dm^3^).

The TLC method is often used in the analysis of compounds with pharmacological significance due to its simplicity, low cost, and speed of analysis. The disadvantage of chromatographic methods is that substances must be isolated from biological material and separated. Until now, only one paper concerning determination of valproic acid in plasma by TLC has been published in the available scientific literature [5]. There are no studies of the use of TLC in the quantitative analysis of valproic acid in pharmaceutical preparations. The Polish Pharmacopoeia VIII in monographs on valproic acid describes only the study of its identity using thin-layer chromatography [44]. Methanol is the solvent used for the study. A mixture of ethyl ether and methylene chloride at a volume ratio of 50:50 (*v/v*) is applied as mobile phase. Bromocresol green is used for detection [44]. The Polish Pharmacopoeia VIII recommends potentiometric titration to quantitative determination of valproic acid [44], whereas the American Pharmacopoeia recommends gas chromatography for the quantification of valproic acid [45].

Corti et al. [5] extracted valproic acid from plasma in an acidic condition, which ensures selectivity. Valproic acid was derivatized in a buffer solution made by mixing potassium dihydrogen phosphate with disodium hydrogen phosphate in 200 mL of water. Reagents used for derivatization were 4-bromophenacyl bromide and 2-naphtacyl bromide. Derivatized valproic acid was analyzed on TLC and HPTLC RP8 plates and HPTLC silica gel 60F_254_ plates, respectively. Three different mobile phases were used: chloroform–cyclohexane (2:1, *v/v*) for NP-HPTLC, ethanol–water (1:0.4, *v/v*) for RP-HPTLC and ethanol–water (1:0.6, *v/v*) for RP-TLC. Densitometric analysis was performed at 280 nm and 254 nm for the naphthyl derivative and phenacyl derivative, respectively. The lower limit of detection of valproic acid in plasma was 9.70 µg/mL and 4.87 µg/mL in NP-TLC and RP-HPTLC, respectively [5].

The aim of this work was to develop the TLC-densitometric method for the determination of valproic acid in capsules of the Convulex pharmaceutical preparation.

## 2. Results and Discussion

### 2.1. Detection of Valproic Acid on a Thin Layer 

**Figure 2:**
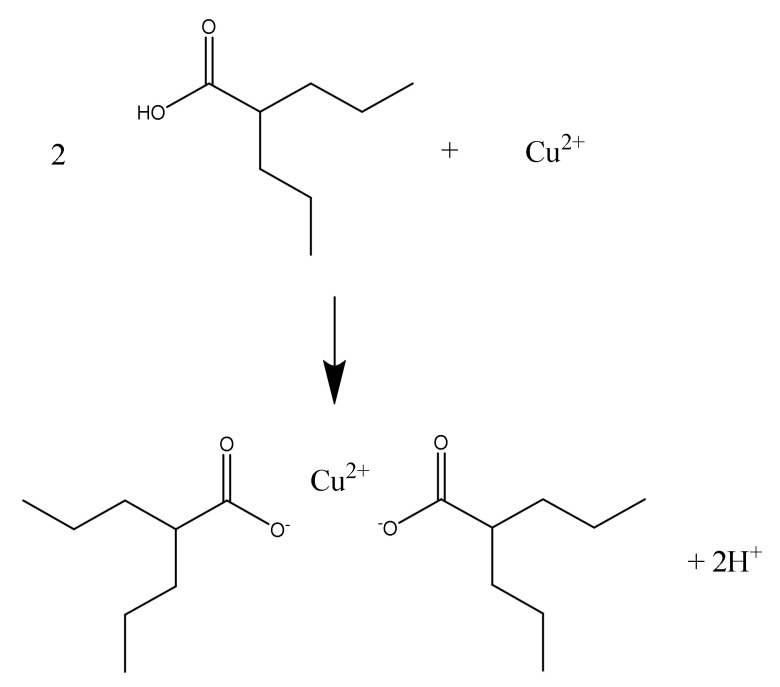
Detection scheme of valproic acid using CuSO_4_ solution.

Several potential reagents for the detection of valproic acid on a thin layer were examined. The usefulness of bromocresol green, which is recommended by pharmacopoeial elaborations [44] for study the identity of valproic acid, was assessed. It has been shown that bromocresol green can be used for qualitative analysis of valproic acid, but it is not suitable for quantitative analysis. The photo (Figure S1) of the plate after detection with bromocresol green and the obtained results of the densitometric analysis (Figure S2) are included in the Supplementary Materials. The valproic acid derivatization method used by Corti et al. [5] is not described precisely, which means that the experiment could not be repeated. The method of valproic acid detection with the use of an aqueous CuSO_4_ solution proposed in our work does not require valproic acid derivatization, which shortens the analysis time. The 2′,7′-dichlorofluorescein-aluminum chloride-iron (III) chloride system for the detection of valproic acid also turned out to be useful, not by spraying the plates, as reported by Dudziński [46], but by dipping the chromatographic plates into individual solutions. Spraying the plates gave heterogeneous chromatographic spots (Figures S3 and S4), while dipping them into solutions gave contrasting spots against the background (Figure S5). However, blurring of the spots at high concentrations of valproic acid was observed, which in turn leads to tailing of the peaks on the densitogram (Figure S6). Therefore, the detection of valproic acid using the 2′,7′-dichlorofluorescein-aluminum chloride-iron (III) chloride system should be recommended for lower concentrations of valproic acid in quantitative analysis, compared to the detection of valproic acid with the use of CuSO_4_. When detecting valproic acid on chromatographic plate using CuSO_4_ solution, copper valproate is most likely formed, according to Figure 2:

When detecting valproic acid on chromatographic plate with the 2′,7′-dichlorofluorescein-aluminum chloride-iron (III) chloride system, the interactions are difficult to determine due to the complexity of the visualizing reagent.

### 2.2. Selectivity

**Figure 3:**
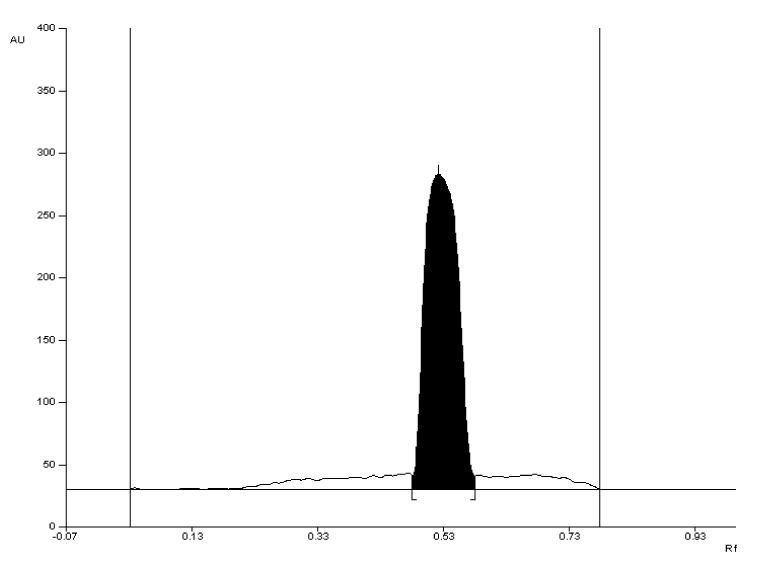
The densitogram of valproic acid standard with R_F_ = 0.57 ± 0.05 after detection with 2% CuSO_4_ solution (detection A).

**Figure 4:**
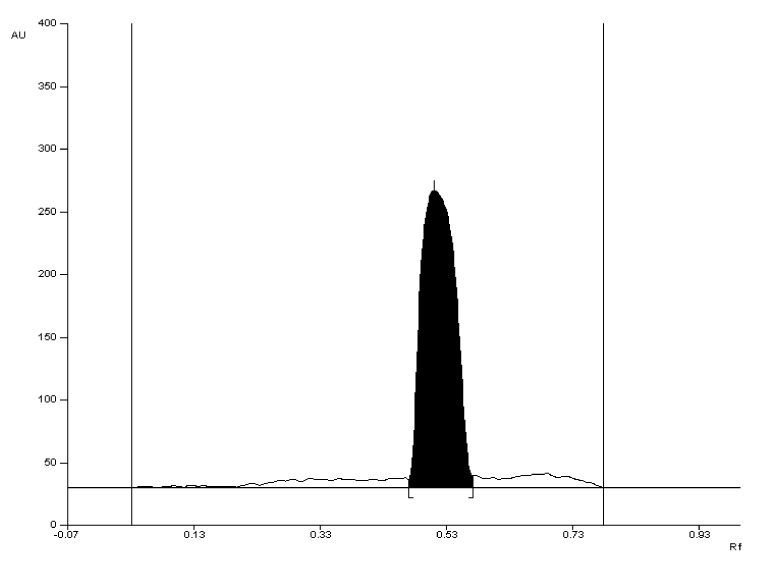
The densitogram of valproic acid coming from the drug with R_F_ = 0.57 ± 0.05 after detection with 2% CuSO_4_ solution (detection A).

**Figure 5:**
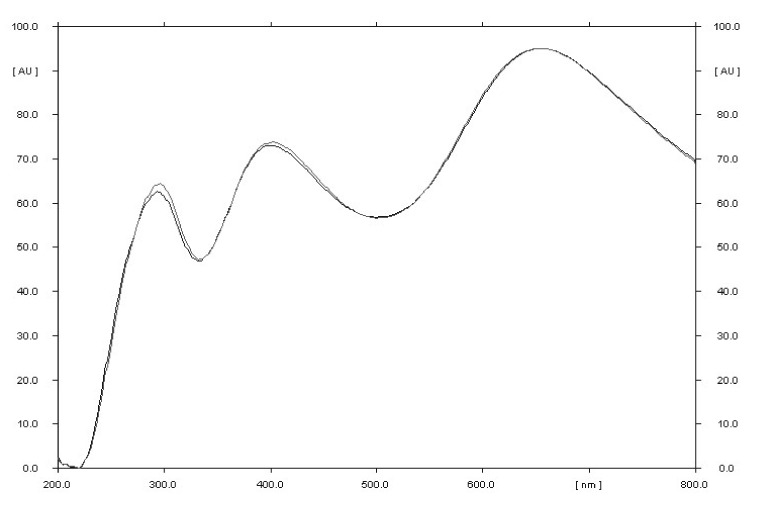
Comparison of the spectrodensitograms of valproic acid standard and valproic acid coming from the drug after detection with 2% CuSO_4_ solution (detection A).

**Figure 6:**
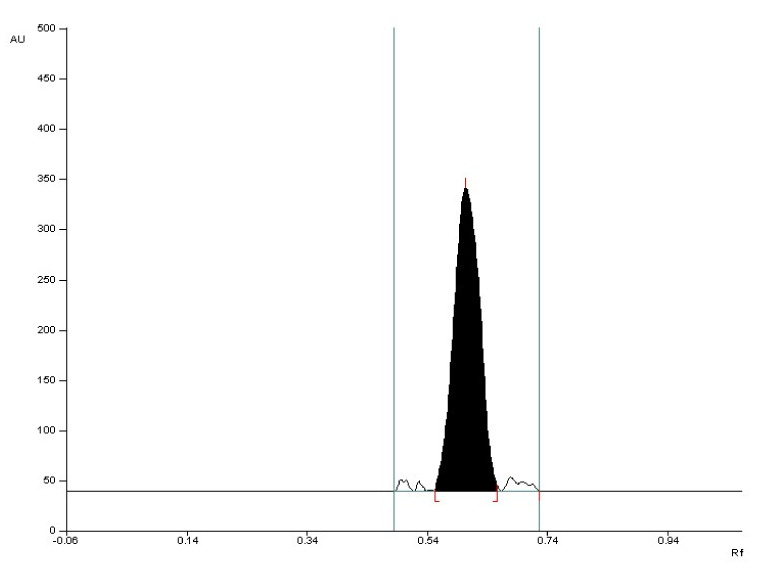
The densitogram of valproic acid standard with R_F_ = 0.57 ± 0.05 after detection with 2′,7′-dichlorofluorescein-aluminum chloride-iron (III) chloride system (detection B).

**Figure 7:**
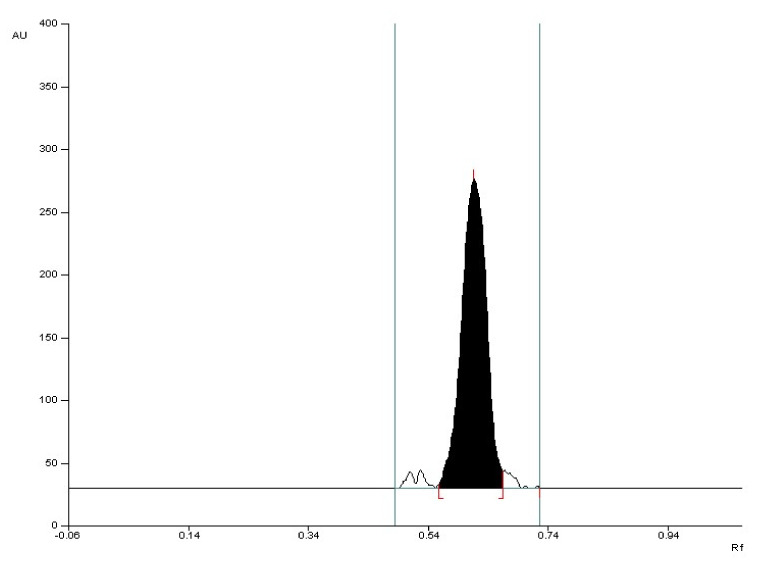
The densitogram of valproic acid coming from drug with R_F_ = 0.57 ± 0.05 after detection with 2′,7′-dichlorofluorescein-aluminum chloride-iron (III) chloride system (detection B).

**Figure 8:**
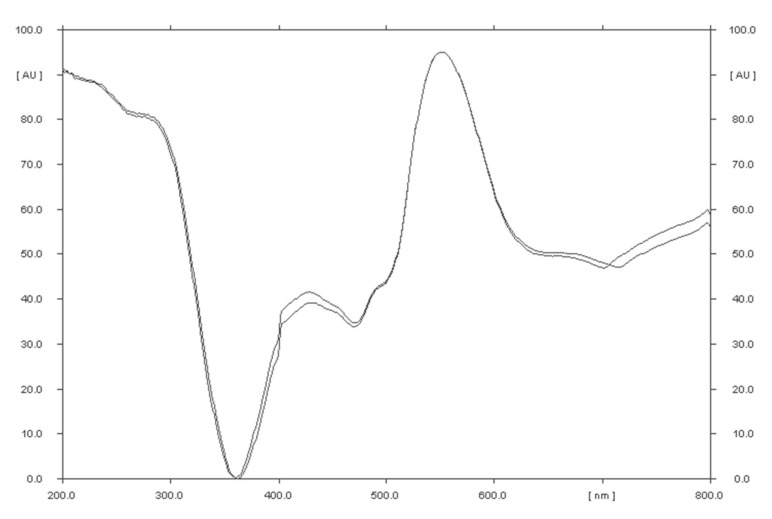
Comparison of the spectrodensitograms of valproic acid standard and valproic acid coming from the drug after detection with 2′,7′-dichlorofluorescein-aluminum chloride-iron (III) chloride system (detection B).

The selectivity of the method was established by comparing the R_F_ values of the valproic acid standard and valproic acid coming from Convulex drug samples and their spectrodensitograms. The R_F_ values of the valproic acid standard and valproic acid coming from Convulex drug samples were 0.57 ± 0.05 in each case. Compatibility in R_F_ values (Figures 3, 4, 6 and 7) was obtained (R_F_ is always equal to 0.57 ± 0.05) and in the spectra of valproic acid standard and valproic acid coming from the Convulex drug (Figures 5 and 8) detected on the chromatographic plate by both detection methods, i.e., A (valproic acid detected using 2% CuSO_4_ solution) and B (valproic acid detected with 2′,7′-dichlorofluorescein-aluminum chloride-iron [III] chloride). It can be concluded that TLC technique combined with densitometric analysis can be used for the quantitative determination of valproic acid in pharmaceutical preparations by analyzing the densitogram of valproic acid coming from Convulex capsules and comparing their spectra. There are no noticeable impurities in the analysis, and the quantitative analysis is based on the measurement of the area of the chromatographic bands. Densitograms and spectrodensitograms are shown in Figure 3, Figure 4, Figure 5, Figure 6, Figure 7 and Figure 8. Thin layer chromatography combined with densitometry is highly selective for the determination of valproic acid in capsules.

The results of the validation of the TLC combined with densitometry are presented in Table 1 and Table 2.

### 2.3. Linearity 

The linearity of the method was determined by measuring the peak area of the chromatographic bands (Tables S1 and Table S2). The linear range was defined between the area of the spots [AU] and the concentration of the valproic acid standard solutions [µg/spot]. Standard concentrations of valproic acid are in the linear range from 20–80 µg/spot (in the case of valproic acid detected with 2% CuSO_4_ solution) and from 1.0–2.0 µg/spot (after detection with the 2′,7′-dichlorofluorescein-aluminum chloride-iron [III] chloride system). Calibration curves are presented in Figures S8 and S9. These results confirm linearity of obtained calibration plots. The adopted TLC-densitometric method allows to quantity of valproic acid in two concentration ranges.

### 2.4. Precision

The precision of the method was determined as the coefficient of variation CV (%) based on the data from the measurement of the valproic acid chromatographic bands. Coefficients of variation for intraday and interday precision ranged from 1.18–2.48% and 1.16–2.89% for valproic acid detected with 2% CuSO_4_ solution, respectively. However, the coefficients of variation for intraday and interday precision ranged from 0.99%–2.22% and 1.86%–2.08% for valproic acid detected using the 2′,7′-dichlorofluorescein-aluminum chloride-iron (III) chlorid system, respectively. The value of the coefficient of variation was < 3% in all cases, which allows to conclude that the analytical method is precise in the quantitative determination of valproic acid in capsules.

### 2.5. Accuracy

The accuracy of the method was evaluated by the measurement of recovery by adding 80%, 100%, and 120% of the valproic acid standard to the drug samples, respectively. The value of the coefficient of variation CV (%) was <1% and <2% in the case of valproic acid detected with 2% CuSO_4_ solution and 2′,7′-dichlorofluorescein-aluminum chloride-iron (III) chlorid system, respectively. The value of coefficient of variation was <2% in each case, which allows to state that the analytical method is accurate in the quantitative determination of valproic acid in capsules.

### 2.6. Limit of Detection (LOD) and Limit of Quantification (LOQ)

The calculated limits of detection and quantification (Table 1 and Table 2) indicate that more sensitive reagent for visualizing valproic acid is the 2′,7′-dichlorofluorescein-aluminum chloride-iron (III) chloride system in relation to the 2% CuSO_4_ solution. 

### 2.7. Robustness

Table 3 shows the results of the robustness of the method for the five changed chromatographic parameters. The coefficients of variation (CV, %) of chromatographic peak areas of valproic acid were ≤1.12% when changing each of the chromatographic parameters. This indicates that the method is robust regardless of the way of valproic acid detection used (detection A and B).

### 2.8. Quantitative Determination of Valproic Acid in Capsules

Valproic acid content in the capsules was calculated using the calibration curve equations presented in Table 1 and Table 2. The statistical data are summarized in Table 4. It was stated that valproic acid contents in capsules, which were determined by the TLC-densitometric method, and using aluminum silica gel plates and acetone–water–chloroform–ethanol–ammonia (30:1:8:5:11) mobile phase and using 2% CuSO_4_ solution and also 2′,7′-dichlorofluorescein-aluminum chloride-iron (III) chloride as a visualizing reagents, were equal to 96.2% and 97.0% in the relation to label claim, respectively. According to American Pharmacopeia the content of valproic acid in capsules should be in the range 90.0–110.0% [45]. Thus, the determined valproic acid contents are within the range given in the Pharmacopoeial monograph. The statistical data comparing the two valproic acid detection methods indicate that they can be used interchangeably depending on the amount of valproic acid in the sample. The coefficients of variance were smaller than 3% in each case. High reproducibility and insignificant differences between the two compared methods were obtained at the 95% probability level for t-test and F-test of significance of 0.893 < 2.101 and 2.39 < 3.18.

### 2.9. Comparison of the Limit of Detection of Valproic Acid Obtained in This Work with Literature Methods

The limit of detection of analyzed valproic acid in this work by TLC technique was compared with the available literature data, in which valproic acid was also determined using various analytical methods. This comparison is summarized in Table 5. This table shows the limits of detection of valproic acid in units in accordance with the cited publications and converted to mol/dm^3^. When comparing the limits of detection in mol/dm^3^, it should be stated that the lowest LOD value (0.75 × 10^−9^ mol/dm^3^) was obtained by Bousquet et al. [14], who used the HPLC method with coulometric electrochemical detection. The limits of detection of valproic acid obtained in this study have higher values than those cited in this table. The values of the limit of detection of valproic acid obtained in this work by TLC using the 2′,7′-dichlorofluorescein-aluminum chloride-iron (III) chloride system have similar values to the limit of detection values obtained during the determination of valproic acid in pharmaceutical preparations using the RP-HPLC [20] and square wave voltammetry [41].

## 3. Materials and Methods

### 3.1. Preparation of Standard Solutions of Valproic Acid 

A standard solution of valproic acid (European Pharmacopoeia (EP) Reference Standard, Sigma-Aldrich, St Louis, MO, USA) at concentration of 100 mg/mL was prepared in acetonitrile. Next, a series of dilutions of valproic acid was prepared to obtain the following concentrations: 90, 80, 70, 60, 50, 40, 30, 20, 15, 10, 7.5, 6.0, 5.0, 4.0, 3.0, 2.0, 1.9, 1.8, 1.7, 1.6, 1.5, 1.4, 1.3, 1.2, 1.1, 1.0, 0.9, 0.8, 0.7 0.6, 0.5, 0.4, 0.3, 0.2, 0.1, 0.08, 0.06, and 0.04 mg/5 mL. The solutions of valproic acid (5 µL) were spotted manually on the chromatographic plates. All chemicals and reagents for TLC method were analytical grade and were purchased from POCh (Gliwice, Poland) or Merck (Darmstat, Germany).

### 3.2. Preparation of the Drug Solution

Two capsules of Convulex 300 mg were cut open and their contents were transferred to a crystallizer. Next, the capsules were rinsed with acetonitrile (solvent). The content of the crystallizer was filtered to a volumetric flask (50 mL), rinsed thoroughly with acetonitrile and replenished with the use of the same solvent to demanded volume. The obtained solution had a concentration of 60 mg/5 mL. Next, the obtained solution was diluted to receive the appropriate concentrations: 40 mg/5 mL, 20 mg/5 mL, 1.2 mg/5 mL, 1.5 mg/5 mL, and 1.8 mg/5 mL. The 5 µL of each of these solutions was spotted onto the chromatographic plates. 

### 3.3. TLC Conditions

The determination of valproic acid was carried out on aluminum silica gel 60F_254_ plates (20 × 10 cm). Before use, the plates were activated at 120 °C for 30 min. The standard and drug solutions in the amount of 5 µL were spotted onto the chromatographic plates. 

The following mobile phases were studied:

(1) *n*-hexane-acetone (4:1);
(2) acetone-water-chloroform-ethanol-ammonia (30:1:3:5:11);
(3) chloroform-cyclohexane (2:1);

and phase (2) modifications, namely:

(2a) acetone-water-chloroform-ethanol-ammonia (20:1:3:5:11);
(2b) acetone-water-chloroform-ethanol-ammonia (30:1:5:5:11);
(2c) acetone-water-chloroform-ethanol-ammonia (30:1:3:1:11);
(2d) acetone-water-chloroform-ethanol-ammonia (30:1:8:5:11).

The 2d mobile phase was chosen as the best and was used for further study. Using this mobile phase, compact chromatographic bands were obtained (Figure 3, Figure 4, Figure 7, Figure 8, Figure S1, Figure S3 and Figure S5).

The plates were developed vertically at room temperature (20 °C) to a distance of 7.5 cm and then dried for 24 h at room temperature (20 °C) in a fume cupboard.

Two different methods of valproic acid detection were used in the work. Namely, the chromatographic plates were immersed for 5 s to:

(A) 2% aqueous CuSO_4_×5H_2_O solution and dried at 120 °C for 6 min;
(B) The visualizing reagent previously described for free acids detection [46] was also used, namely, 2′,7′-dichlorofluorescein-aluminum chloride-iron (III) chloride system.

Solution I, 0.05% 2′,7′-dichlorofluorescein in ethanol; solution II, 1% ethanolic solution of aluminum chloride; and solution III, 1% aqueous solution of iron(III) chloride, were used as visualizing reagents. Plates were dipped in solution I and dried for 3 min at 100 °C, before being dipped in solution II and dried again for 3 min. Finally, plates were dipped in solution III. 

### 3.4. Densitometric and Spectrodensitometric Analysis

Densitometric and spectrodensitometric measurements were performed using a TLC Scanner 3 (Camag, Switzerland) densitometer. The radiation sources were deuterium and tungsten lamps. The spectrodensitometric analysis was performed in wavelength range from 200 to 800 nm. The scanning speed was 20 mm/s, the slit dimension was 12 × 0.4 mm, and the data resolution was 100 µm/step. Densitometric scanning of plates with this densitometer and winCATS 1.4.2 software was performed at the optimal wavelength of 650 nm and 550 nm, after CuSO_4_×5H_2_O detection and the 2′,7′-dichlorofluorescein-aluminum chloride-iron (III) chloride system, respectively.

### 3.5. Validation of TLC Method

The proposed method was validated by linearity selectivity, intraday and interday precision, accuracy, limit of detection, limit of quantification, and robustness. 

#### 3.5.1. Linearity and Range

The linearity range was evaluated by analysis of standard solutions of valproic acid. The standard solutions (5 µL) were applied to the chromatographic plates activated for 30 min at the temperature of 120 °C. The plates were developed in the chromatographic chamber using acetone-water-chloroform-ethanol-ammonia (30:1:8:5:11 *v/v*/*v/v*/*v*) mobile phase. The chamber was saturated for 20 min. The analysis was repeated three times.

#### 3.5.2. Intraday and Interday Precision

The precision of the method was verified by the analysis of three solutions of drug at the following concentrations: 20 mg/5 mL, 40 mg/5 mL, and 60 mg/5 mL (in the case of valproic acid detected using 2% CuSO_4_ solution), and 1.2 mg/5 mL, 1.5 mg/5 mL, and 1.8 mg/5 mL (in the case of valproic acid detected by 2′,7′-dichlorofluorescein-aluminum chloride-iron [III] chloride system). The intraday and interday precision was evaluated as the relative standard deviation (coefficient of variation, CV [%]).

#### 3.5.3. Accuracy 

The accuracy of the method was evaluated by the measurement of recovery. Three solutions were prepared with the addition of 80%, 100%, and 120% of the standard.

#### 3.5.4. Limit of Detection (LOD) and Limit of Quantification (LOQ)

Limit of detection and quantification was determined by preparing three standard solutions of valproic acid at concentration of 15, 10, and 7.5 mg/5 mL (in the case of valproic acid detected with 2% CuSO_4_ solution), and three standard solutions of valproic acid at concentration of 1.0, 0.9, and 0.8 mg/5 mL (in the case of valproic acid detected by the 2′,7′-dichlorofluorescein-aluminum chloride-iron [III] chloride system). The LOD and LOQ calculation methodology was given in our earlier works [47,48,49]. The analyses were repeated three times.

#### 3.5.5. Robustness

The robustness of the method was checked by examining how small changes in the chromatographic conditions affect the peak area of sample of valproic acid from the drug. The parameters changed were the acetone content in the composition of the mobile phase (±0.5 mL), the ammonia content in the composition of the mobile phase (±0.2 mL), the volume of the mobile phase used (±5 mL), the activation time of the chromatographic plate (±5 min), and the time of saturation of chromatographic chamber (±3 min). The analyses were repeated five times. Drug solutions at concentrations of 40 mg/5 mL (in the case of valproic acid detected using 2% CuSO_4_ solution) and 1.5 mg/5 mL (in the case of valproic acid detected by the 2′,7′-dichlorofluorescein-aluminum chloride-iron [III] chloride system) were analyzed.

### 3.6. Statistical Analysis

Statistical evaluation of the obtained results was prepared by Statistica v 13.0 PL (StatSoft, Kraków, Poland).

## 4. Conclusions

The developed thin layer chromatographic method combined with densitometric analysis for the determination of valproic acid in *Convulex* capsules turned out to be selective, linear, accurate, precise, and robust. The identity of the drug could be determined by analyzing the R_F_ values and spectrodensitograms of valproic acid from capsules and the valproic acid standard. The calculated content of valproic acid in capsules was within the range given in monographs 34 of the US Pharmacopoeia (90.0–110.0%) in relation to label claim. Valproic acid contents in capsules were equal to 96.2% and 97.0% using 2% CuSO_4_ solution and 2′,7′-dichlorofluorescein-aluminum chloride-iron (III) chloride as the visualizing reagents in the relation to label claim, respectively. Therefore, the developed thin layer chromatographic method coupled with densitometric analysis can be successfully applied in the quantitative determination of valproic acid in capsules, both after the detection of valproic acid with CuSO_4_ solution and 2′,7′-dichlorofluorescein-aluminum chloride-iron (III) chloride as the visualizing reagents. Thin layer chromatography is distinguished by its simplicity, speed, and low cost compared to other methods for the determination of valproic acid. A great advantage of the developed conditions for the determination of valproic acid is the fact that there is no need to derivatize valproic acid before its analysis, which significantly shortens the analysis time. Chromatographic techniques (LC, HPLC, GC) prevail in the publications on the analysis of valproic acid described in the scientific literature. Taking into account the limit of detection values, the TLC method in combination with densitometry is comparable to RP-HPLC and square wave voltammetry methods and can be successfully used for the analysis of valproic acid in capsules, especially when there is no other analytical techniques available. The advantages of TLC are simplicity, low cost, and fast analysis results.

## Figures and Tables

**Table 1:** Method-validation data for the quantitative determination of valproic acid by NP-TLC with densitometry using 2% solution of CuSO_4_ as visualizing reagent.

Method Characteristic	Results
Retardation factor (R_F_)	0.57 ± 0.05
Range [μg/spot]	20.0–80.0
Linearity [μg/spot]	A = 105.0(±5.1)x + 971.5(±273.2) *n* = 7; r = 0.994; s = 268.4; F = 428; *p* < 0.0001
Limit of Detection (LOD) [(g/spot]	5.8
Limit of Quantification (LOQ) [(g/spot]	17.4
	For capsules
Accuracy	
for 80% valproic acid added (*n* = 6)	R = 97.8%; CV = 0.81%
for 100% valproic acid added (*n* = 6)	R = 98.2%; CV = 0.61%
for 120% valproic acid added (*n* = 6)	R = 99.0%; CV = 0.67%
Precission (CV, [%])	
Intraday	
for 60.0 (g/spot (*n* = 3)	1.18
for 40.0 (g/spot (*n* = 3)	2.48
for 20.0 (g/spot (*n* = 3)	2.23
Interday	
for 60.0 (g/spot (*n* = 3)	1.16
for 40.0 (g/spot (*n* = 3)	2.19
for 20.0 (g/spot (*n* = 3)	2.89
Robustness (CV, [%])	robust

x-amount [μg/spot] of drug analyzed, r-correlation coefficient, R-recovery [%], CV-coefficient of variation [%].

**Table 2:** Method-validation data for the quantitative determination of valproic acid by NP-TLC with densitometry using 2′,7′-dichlorofluorescein-aluminum chloride-iron (III) chloride as visualizing reagent.

Method Characteristic	Results
Retardation factor (R_F_)	0.57 ± 0.05
Range [μg/spot]	1.0–2.0
Linearity [μg/spot]	A = 6883.6(±34.2)·x − 979.9(±308.8) *n* = 11; r = 0.996; s = 211.3; F = 1167; *p* < 0.0001
Limit of Detection (LOD) [(g/spot]	0.32
Limit of Quantification (LOQ) [(g/spot]	0.97
	For capsules
Accuracy	
for 80% valproic acid added (*n* = 6)	R = 101.1%; CV = 1.11%
for 100% valproic acid added (*n* = 6)	R = 99.3%; CV = 1.38%
for 120% valproic acid added (*n* = 6)	R = 99.8%; CV = 1.98%
Precission (CV, [%])	
Intraday	
for 1.2 (g/spot (*n* = 3)	0.99
for 1.5 (g/spot (*n* = 3)	1.87
for 1.8 (g/spot (*n* = 3)	2.22
Interday	
for 1.2 (g/spot (*n* = 3)	2.08
for 1.5 (g/spot (*n* = 3)	1.99
for 1.8 (g/spot (*n* = 3)	1.86
Robustness (CV, [%])	robust

**Table 3:** Robustness of the proposed method for the determination of valproic acid in the drug using 2% CuSO_4_ solution (detection A) and 2′,7′-dichlorofluorescein-aluminum chloride-iron (III) chloride as visualizing reagent (detection B).

Chromatographic Conditions Changed	Detection Method			
	A		B	
	%CV	% Assay of Valproic Acid	%CV	% Assay of Valproic Acid
Acetone content (±0.5 mL)	1.08	95.5	1.12	97.5
Ammonia content (±0.2 mL)	0.84	96.4	0.78	96.9
Mobile phase volume (±5 mL)	0.99	95.9	0.95	97.9
Time of activation of chromatographic plate (±5 min)	0.56	96.5	0.78	96.5
Time of saturation of chromatographic chamber (±3 min)	0.96	96.7	0.95	98.1

**Table 4:** Valproic acid assay [mg/capsule] obtained from ten repeated different analysis of by TLC-densitometric using A and B detection methods.

No.	Assay Using Detection Method	
	A	B
1	276.4	289.6
2	295.8	295.4
3	281.6	288.8
4	285.7	284.8
5	283.1	286.0
6	294.8	293.9
7	289.7	291.7
8	296.8	299.1
9	288.5	292.3
10	293.7	287.8
Average	288.6	290.9
Label claimed	300	300
Amount of valproic acid (%) in relations to the label claim	96.2%	97.0%
Standard deviation (SD)	6.84	4.42
Coefficient of variation [CV, %]	2.37	1.52
Comparison of detection methods A and B		
*t* test	t calculated	0.893
	t_(95%.18)_ tabulated	2.101
test	F calculated	2.39
	F_(95%_.*_f_*_1 =* f*2 = 9)_ tabulated	3.18

**Table 5:** Comparison of the limit of detection of valproic acid determined in pharmaceutical preparations.

Analytical Method	LOD in the Unit		Refs.
	According to the Literature Data	Converted to mol/dm^3^	
HPLC with coulometric electrochemical detection	0.75 pmol/mL	0.75 × 10^−9^	[14]
RP-HPLC	5.4411 µg/mL	3.77 × 10^−5^	[17]
RP-HPLC	30.38 µg/mL	1.82 × 10^−4^	[20]
HPLC	6.8 µg/mL	4.72 × 10^−5^	[23]
GC-FID	0.8 µg/mL	5.55 × 10^−6^	[35]
GC-FID	0.05 µg/mL	3.47 × 10^−7^	[37]
Square wave voltammetry	21.05 µg/mL	1.46 × 10^−4^	[41]
TLC with using CuSO_4_ for detection	5.8 µg/spot	8.04 × 10^−3^	in this work
TLC with using a 2′,7′-dichlorofluorescein-aluminum chloride-iron (III) chloride for detection	0.32 µg/spot	4.44 × 10^−4^	in this work

## Data Availability

Not applicable.

## Supplementary Materials

The following are available online. Figure S1: Valproic acid after detection with an aqueous solution of bromocresol green; (a) dipping the plate into the solution (immediately after immersion); and (b) immersing the plate into the solution (after 6 min of drying at 120 °C). Figure S2: Densitogram of valproic acid after detection with bromocresol green. Figure S3: Valproic acid after detection by spraying with 2′,7′-dichlorofluorescein-aluminum chloride-iron (III) chloride system as a visualizing reagent. Figure S4: Densitogram of valproic acid after detection by spraying with 2′,7′-dichlorofluorescein-aluminum chloride-iron (III) chloride system as the developing reagent. Figure S5: Valproic acid after detection by immersion of the chromatographic plate in the components of the system 2′,7′-dichlorofluorescein-aluminum chloride-iron (III) chloride. Figure S6: Densitogram of valproic acid after detection by immersion of the chromatographic plate in the components of the system 2′,7′-dichlorofluorescein-aluminum chloride-iron (III) chloride (fourth lane in the above photo). Figure S7: Valproic acid after detection with 2% aqueous solution of CuSO_4_ by immersion of the plate. Table S1: Results of densitometric analysis of standard solutions of valproic acid (mean from *n* = 3) after detection with 2% aqueous solution of CuSO_4_ by immersion of the plate. Figure S8: Calibration curve of valproic acid after detection with 2% aqueous solution of CuSO_4_ by immersion of the plate. Table S2: Results of densitometric measurements of standard solutions of valproic acid (mean from *n* = 3) after detection by immersion of the chromatographic plate in the components of the system 2′,7′-dichlorofluorescein-aluminum chloride-iron (III) chloride. Figure S9: Calibration curve of valproic acid after detection by immersion of the chromatographic plate in the components of the system 2′,7′-dichlorofluorescein-aluminum chloride-iron (III) chloride.

## Sample Availability

Samples of the compounds are available from the authors.
