# Supplementary material for: Use of TLC-Densitometric Method for Determination of Valproic Acid in Capsules
Source: Molecules. 2022 Jan 24;27(3):752. doi: 10.3390/molecules27030752 (PMC8839749; doi:10.3390/molecules27030752)
Supplement: Supplementary file 1 [file molecules-27-00752-s001.zip › molecules-1571098-supplementary.pdf]

Supplementary Materials

Use of TLC-densitometric Method for Determination of Valproic Acid in Capsules

Wioletta Parys* **and Alina Pyka-Pająk***

Department of Analytical Chemistry, Faculty of Pharmaceutical Sciences in Sosnowiec, Medical University of Silesia in Katowice, Jagiellońska 4, 41-200 Sosnowiec, Poland
*Correspondence: wparys@sum.edu.pl (W.P.); apyka@sum.edu.pl (A.P.-P.); Tel.: +48-32-364-15-34 (W.P.); +48-32-364-15-30 (A.P.-P.)

a) b)

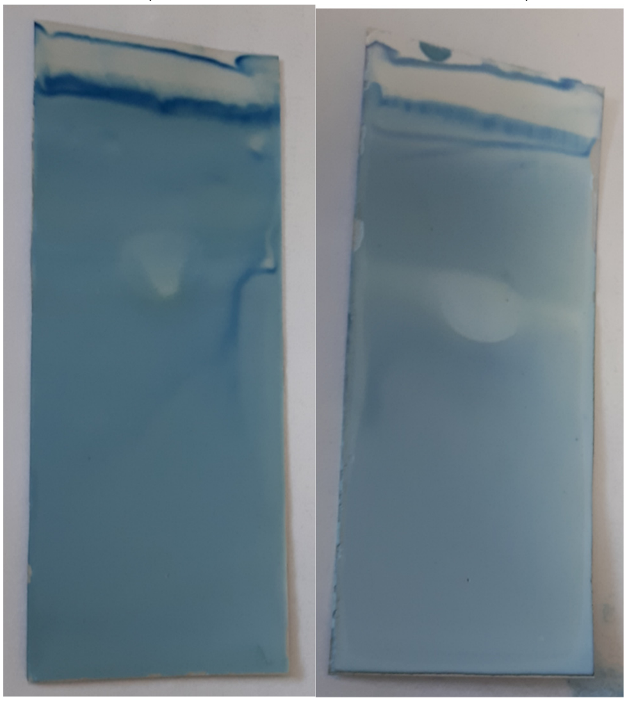

Figure S1: Valproic acid after detection with an aqueous solution of bromocresol green:
(a) dipping the plate into the solution (immediately after immersion)
(b) immersing the plate into the solution (after 6 minutes of drying at 120°C)

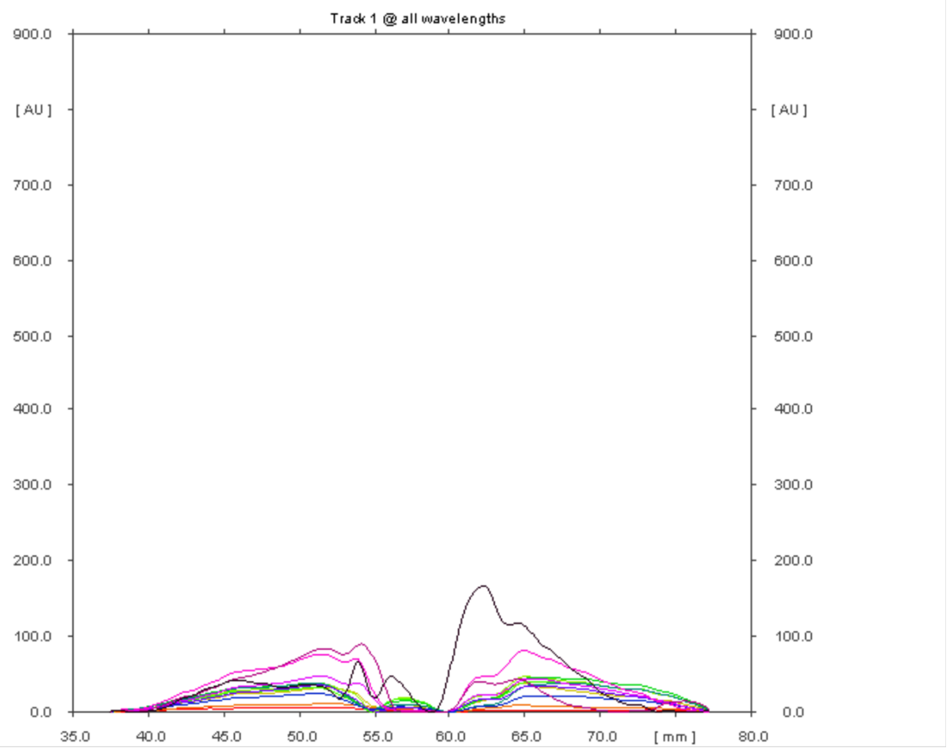

Figure S2: Densitogram of valproic acid after detection with bromocresol green.

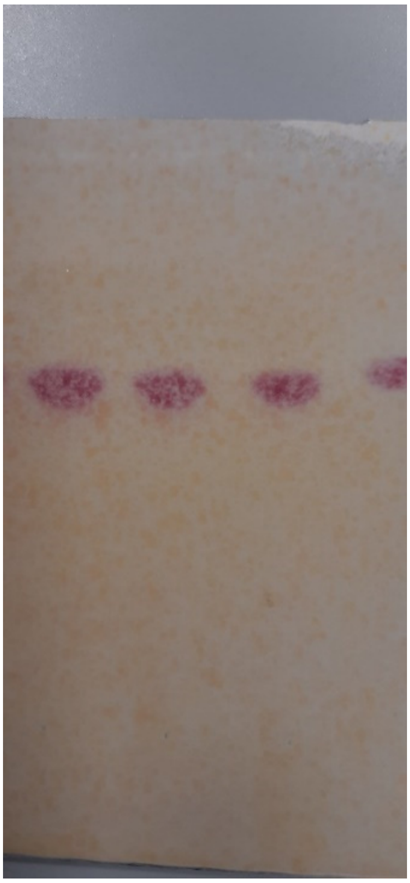

Figure S3: Valproic acid after detection by spraying with 2', 7'-dichlorofluorescein - aluminum chloride-iron (III) chloride system as a visualizing reagent.

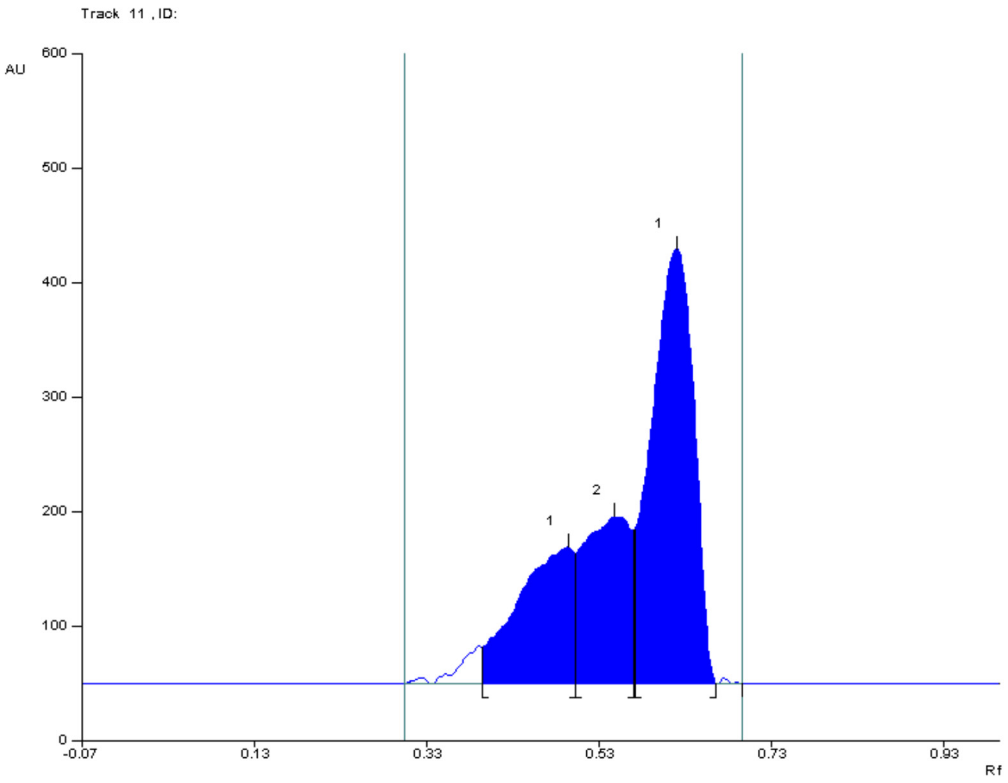

Figure S4: Densitogram of valproic acid after detection by spraying with 2', 7'-dichlorofluorescein - aluminum chloride-iron (III) chloride system as the developing reagent.

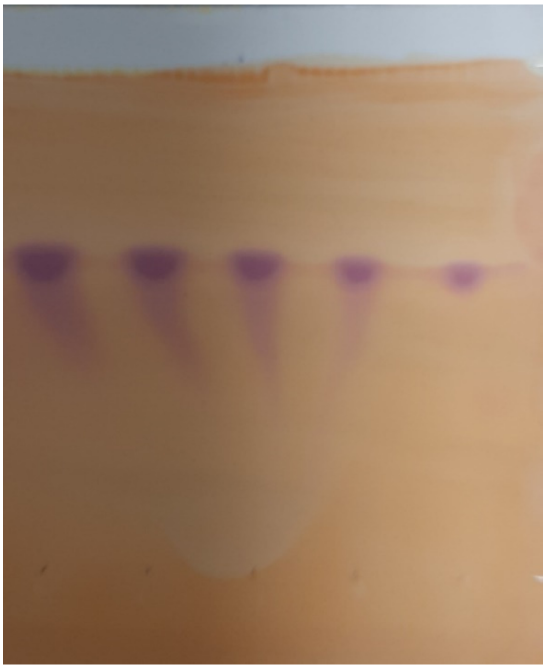

Figure S5: Valproic acid after detection by immersion of the chromatographic plate in the components of the system 2', 7'-dichlorofluorescein - aluminum chloride-iron (III) chloride.

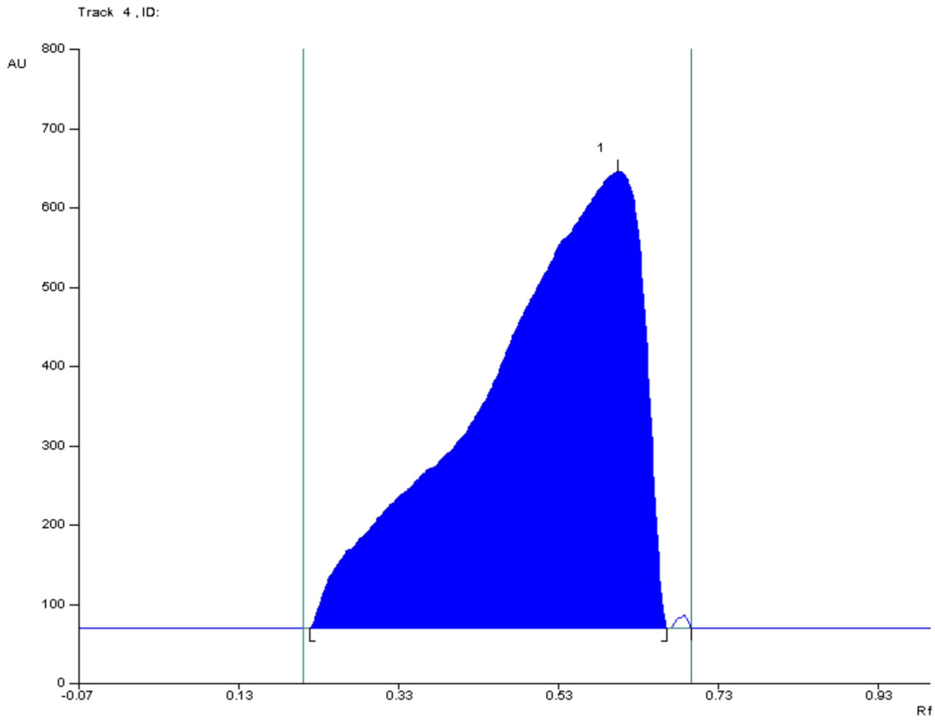

Figure S6: Densitogram of valproic acid after detection by immersion of the chromatographic plate in the components of the system 2', 7'-dichlorofluorescein-aluminum chloride-iron (III) chloride (fourth lane in the above photo).

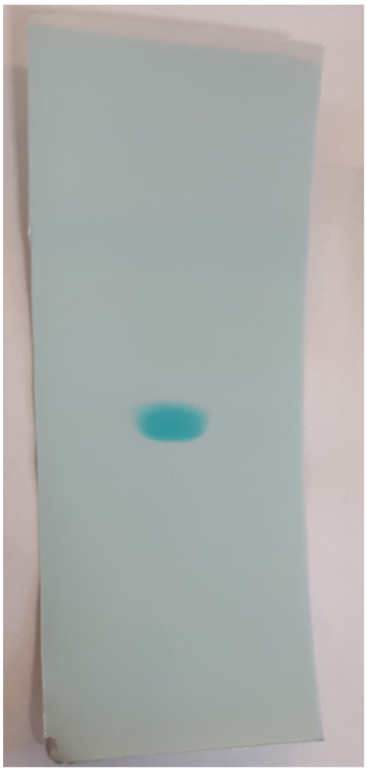

Figure S7: Valproic acid after detection with 2% aqueous solution of $CuSO_4$ by immersion of the plate.

Table S1. Results of densitometric analysis of standard solutions of valproic acid (mean from n = 3) after detection with 2% aqueous solution of $CuSO_4$ by immersion of the plate.

The amount of valproic acid [μg/spot]	Chromatographic band area of spot [AU]
100	10916
90	9195
80	8997
70	8377
60	7584
50	6476
40	5243
30	3955
20	2924

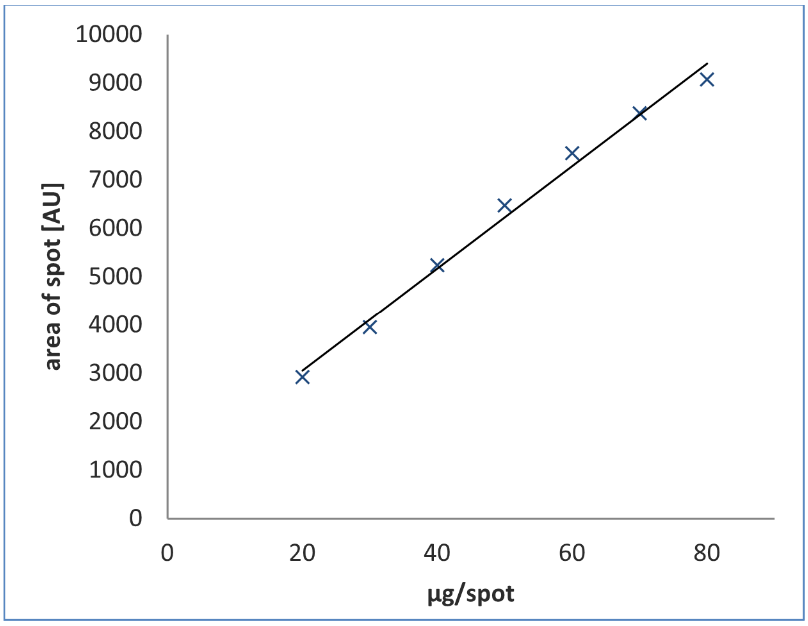

Figure S8. Calibration curve of valproic acid after detection with 2% aqueous solution of $CuSO_4$ by immersion of the plate.

Table S2. Results of densitometric measurements of standard solutions of valproic acid (mean from n = 3) after detection by immersion of the chromatographic plate in the components of the system 2', 7'-dichlorofluorescein - aluminum chloride-iron (III) chloride.

The amount of valproic acid [μg/spot]	Chromatographic band area of spot [AU]
2.0	13224
1.9	11985
1.8	11089
1.7	10725
1.6	9858
1.5	9423
1.4	8625
1.3	8023
1.2	7255
1.1	6485
1.0	6108

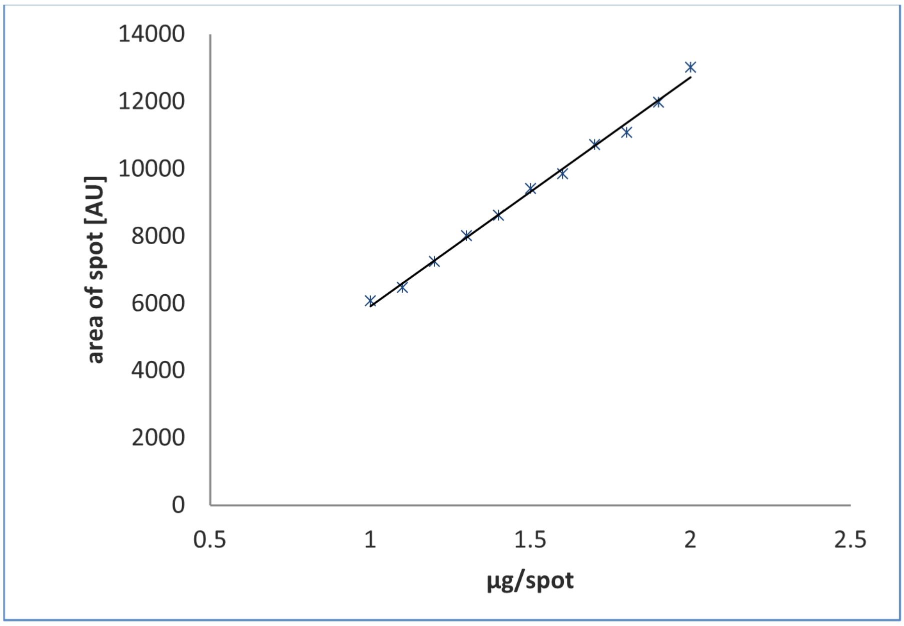

Figure S9. Calibration curve of valproic acid after detection by immersion of the chromatographic plate in the components of the system 2', 7'-dichlorofluorescein - aluminum chloride-iron (III) chloride.
